# Demographics, Clinical Content, Use Patterns, and Care-Seeking Intent Across Two Generations of AI-Enabled Clinical Triage Tools (A Traditional Structured Questionnaire and a Large Language Model–Enabled Conversational Interface): Comparative Retrospective Observational Study

**DOI:** 10.2196/84469

**Published:** 2026-09-22

**Authors:** Maria Marecka, Anna Nowicka, Aleksandra Suwińska, Piotr Orzechowski

**Affiliations:** 1 Infermedica Inc. Denver, CO United States; 2 Infermedica Wroclaw Poland; 3 Department of Human Morphology and Embryology Division of Histology and Embryology Wroclaw Medical University Wroclaw Poland

**Keywords:** digital front door, virtual triage, clinical decision support systems, artificial intelligence, AI, natural language processing, large language models, patient engagement, adherence and compliance

## Abstract

**Background:**

Virtual clinical triage is central to digital front door strategies, yet how interaction mode—structured, closed-ended AI questionnaires vs large language model (LLM)–enabled conversational dialogue—affects information capture, engagement, and alignment with care recommendations is unclear.

**Objective:**

This study compared demographics, clinical content, engagement, and alignment with recommended care between a traditional, structured questionnaire interface (traditional triage [TT]) and an LLM-enabled conversational interface (conversational triage [CT]) sharing the same validated Bayesian reasoning engine.

**Methods:**

This comparative, retrospective observational study analyzed 116,890 encounters over 28 weeks (January 2025 to August 2025) completed on a virtual triage website. Users self-selected TT or CT. Primary end points included demographic and clinical characteristics, engagement patterns, and self-reported intended adherence to triage recommendations. Analyses used poststratification weighting by age and sex. Group differences were assessed with the chi-square test (with Rao-Scott correction for weighted data) and survey-weighted Wilcoxon rank-sum test; *P* values for comparisons of proportions were adjusted for multiple comparisons using the Benjamini-Hochberg false discovery rate procedure. For CT, opening and closing sentiment (positive, neutral, or negative) was labeled using a prompt in Gemini 2.5 Flash.

**Results:**

Of 116,890 respondents, 100,533 (86%) used TT and 16,357 (14%) used CT. Female users were the majority in both groups but less predominant in CT (10,402/16,357, 64% vs 71,039/100,533, 71%) than in TT. TT users skewed younger (aged 18-29 years >50%), whereas CT was more evenly distributed with higher shares at ages 12 to 17, 30 to 44, and ≥45 years. Median session duration was longer with CT than with TT (8 minutes 21 seconds, IQR 6 minutes 10 seconds to 11 minutes 51 seconds vs 4 minutes 25 seconds, IQR 3 minutes 14 seconds to 6 minutes 22 seconds, respectively). CT elicited more clinical findings (median 36, IQR 31-43 vs 32, IQR 27-38; *P*<.001) and surfaced more mental health evidence (eg, depressive symptoms, 8.5% vs 6.2%; *P*<.001). Posttriage intent survey completion was higher with CT (5104/16,357, 31.2% vs 5968/100,533, 5.9%). Self-reported intended adherence to the recommended level of care was higher with CT (1749/5104, 34.3% vs 1740/5968, 29.2%; *P*<.001), especially at extremes of acuity: self-care (534/625, 85.4% vs 325/525, 61.9%), emergency room (171/723, 23.7% vs 100/949, 10.5%), and ambulance (39/339, 11.5% vs 27/554, 4.9%; all *P*<.001). Among CT encounters, positive sentiment increased markedly, while negative sentiment rose slightly.

**Conclusions:**

The LLM-enabled CT was used by a more demographically diverse user base and was associated with richer clinical context, deeper engagement, and higher self-reported intended adherence among survey respondents, particularly for low-acuity self-care and high-acuity emergency scenarios, where reassurance or urgent escalation is critical. This highlights the potential of hybrid LLM tools that integrate validated clinical logic to support engagement, information gathering, and care navigation in digital front door settings.

## Introduction

Health systems are confronting multiple pressures due to rising patient volumes, aging populations, workforce shortages, and increasing complexity of care [1,2]. These pressures stretch clinical capacity and disrupt timely access, motivating the adoption of digital front door strategies, in which virtual clinical triage is an important component [3,4]. By serving as the initial point of contact, virtual triage can efficiently route patients to an appropriate level of care, thereby improving resource allocation, reducing unnecessary clinician burden, and enhancing patient access and satisfaction [3,4]. Current implementations of digital triage support these benefits: such tools have been deployed as front door portals for health systems, and they show potential to align care with acuity needs and prevent avoidable high-intensity service use. For example, in a large Australian health system, virtual triage safely redirected more than half of users who initially intended to attend the emergency room (ER) to lower-acuity services [3]. Similarly, a Portuguese health insurance company reported de-escalation to lower-acuity settings for 48% of users who had planned an ER visit, improving system-level resource allocation [5].

To date, the predominant modality for virtual triage tools has been the structured interview or questionnaire, in which users answer a dynamic sequence of closed-ended questions about their symptoms and risk factors. Such tools combine evidence-based medical knowledge and clinical expertise in a decision-tree or AI-based probabilistic logic that generates triage advice after a structured interview [6,7]. These triage interfaces are widely implemented and effective for care navigation, but their closed-ended format can constrain expression and miss nuance—especially for sensitive or complex concerns. Recent evidence syntheses have also emphasized that both symptom-assessment applications and large language models (LLMs) are increasingly being evaluated for self-triage decisions, but that their accuracy remains variable and context-dependent [8].

Recently, the emergence of a newer generation of AI—generative AI—and, in particular, LLMs has enabled a new modality of AI-driven triage: the conversational dialogue interface. Rather than prompting users with predefined questions and answer options, an LLM-enabled triage system allows patients to describe their concerns in free text, then engages in a dynamic dialogue—asking follow-up questions, interpreting responses, and providing information—in natural, conversational language [9]. LLM-based tools may offer additional advantages: multiple studies have shown that LLM-generated responses to patient questions are often perceived as more empathic and sometimes of higher quality than clinician replies, potentially lowering barriers to disclosure of sensitive information and improving engagement [10-12].

However, LLM-only approaches to medical advice have important safety and transparency challenges. If left unchecked, a generative model may produce inaccurate or misleading health information; this phenomenon, known as AI hallucination, can also introduce biases or harmful advice, and such models often lack explainability or a reliable evidence base for how conclusions are drawn, which can impact safety and user trust [13-16]. Recognizing these risks, developers have increasingly turned to neuro-symbolic or hybrid AI strategies that combine LLMs with evidence-based and clinically validated knowledge-based triage systems. By combining the natural language advantages of LLMs with a deterministic medical inference engine, these systems aim to keep triage outcomes grounded in clinical evidence and established guidelines. The LLM component handles the free-form user interaction—understanding user messages, displaying empathy, and maintaining context—while using the underlying deterministic algorithm for diagnostic or triage decisions.

Despite the theoretical appeal of these LLM-enabled triage interfaces, there is currently a scarcity of real-world evidence on how a conversational tool compares to the traditional structured questionnaire format. Specifically, it is unclear how interface design influences the breadth and depth of clinical information captured, user interaction patterns, and alignment with recommended levels of care. Understanding these differences is crucial, as different interface modalities could affect the clinical effectiveness and user satisfaction of digital triage programs.

To address this evidence gap, we conducted a retrospective comparison of the real-life use of a traditional, structured questionnaire triage (traditional triage; TT) and a novel LLM-based conversational triage (CT) system—both operating on the same clinically validated Bayesian reasoning engine, allowing comparison of the 2 interface modalities that share the same medical logic. By comparing these two triage modalities in a real-world context, this study aimed to describe differences in user experience, information capture, and alignment with care recommendations between a next-generation LLM-enabled conversational interface and a traditional AI questionnaire interface.

## Methods

### Study Design, Participants, Data Source, and Outcomes

This comparative retrospective observational study evaluated all user-initiated virtual clinical triage encounters completed on the public Symptomate (Infermedica) website during the period from January 25, 2025, to August 8, 2025 (inclusive). Symptomate is designed for public use free of charge and is not attached to a specific health system. It is accessed by individuals independently seeking online symptom assessment and triage. During this 28-week period, users could select between two modalities: a longer-standing traditional structured AI questionnaire interface (TT) and an LLM-enabled conversational interface (CT). No incentives were offered beyond the informational value of the tools themselves. In total, 116,890 distinct completed encounters were recorded. Deidentified session logs provided user demographics (age and sex); clinical characteristics (symptoms, signs, and risk factors, distinguishing initial chief complaints from subsequent reported evidence); interaction metrics (time of day in Coordinated Universal Time [UTC] and day of the week at the time of the interview, question count, and session duration); triage outcomes across 5 acuity levels (self-care, routine outpatient consultation, urgent outpatient consultation, ER self-attendance, or ambulance); self-reported posttriage care–seeking intent; and, for CT, sentiment labels for users’ interactions with the tool at the start and end of the encounter.

### The Studied Interfaces

Both interfaces—TT and CT—operate on the same probabilistic Bayesian reasoning engine and clinically validated knowledge base of more than 900 diseases, 1800 symptoms, and 340 risk factors (symbolic reasoning over an explicit, clinically curated knowledge base). As evidence is gathered, the engine dynamically selects the most informative follow-up questions and estimates the likelihood of events, such as the most likely condition and the most appropriate triage level (self-care, routine outpatient consultation, urgent outpatient consultation, ER self-attendance, or ambulance). Users entering the landing page in English could choose between the 2 tools, as shown in Figure 1. The TT interface presents a dynamically ordered, closed-ended question sequence, whereas the CT interface layers an LLM-enabled dialogue over the identical probabilistic core to accept free-text input, clarify terminology, and surface the engine’s reasoning in a conversational manner (Figures 2 and 3, respectively). The LLM serves as a preprocessing layer that extracts symptom-related entities from free text. Entities are then mapped to standardized medical concepts. Although the precision of the extraction and mapping process was not validated in this research, preliminary internal assessments indicated a precision rate of 73% across 2500 user messages; furthermore, benchmarking tests using 120 clinical vignettes demonstrated that the performance of the CT interface was on par with the TT [17]. The TT questionnaire (Medical Guidance Platform: Triage Module; Infermedica) is registered as a class IIb medical device in the European Union and is regulated by the Food and Drug Administration (FDA) as a general wellness product in the United States. The CT system was made available as a noncommercial usability-study version of a precertification interface and was not marketed or deployed as a certified medical device during the study period.

**Figure 1:**
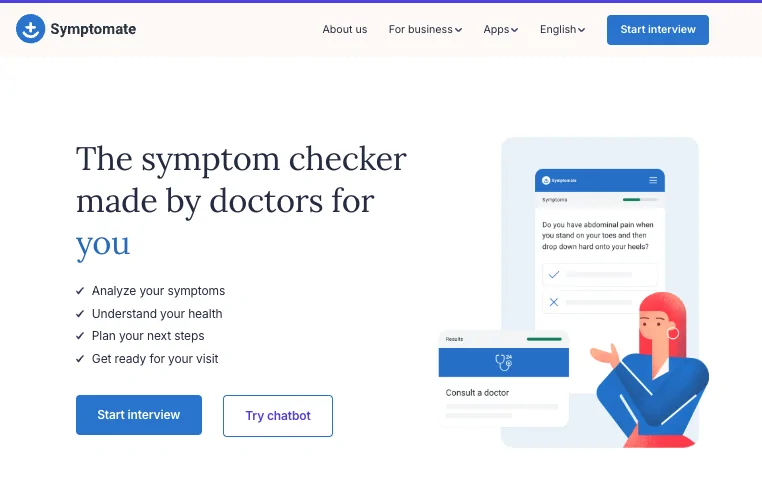
Landing page encouraging users to use traditional triage ("start interview") or conversational triage ("try chatbot").

**Figure 2:**
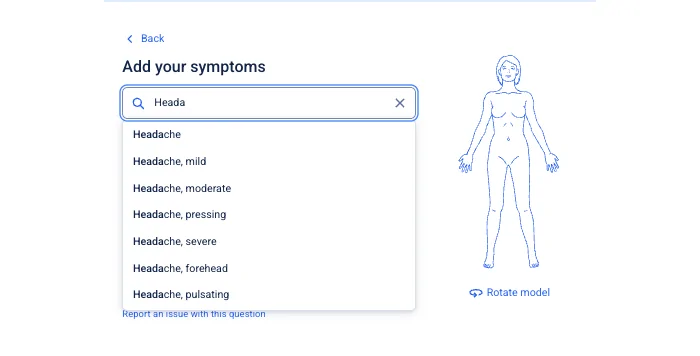
Chief complaint collection interface in traditional triage.

**Figure 3:**
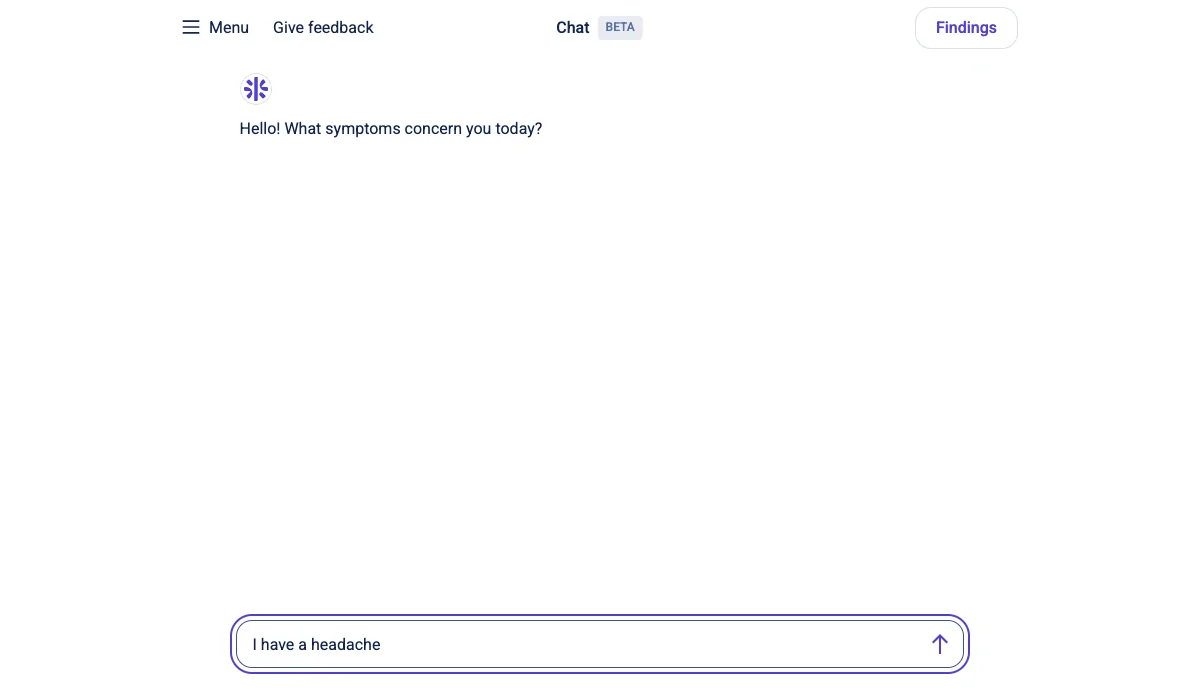
Chief complaint collection interface in conversational triage.

### Statistical Analyses

#### Data Filtering

For inclusion in the study, only users who accessed the Symptomate landing page in English were considered for both tools. For all metrics, only finished interviews were included, meaning that the user was presented with the final triage recommendation.

#### Weighting Method

To ensure an accurate comparison between the two tools, given observed differences in user demographics, a weighting method was applied. Each completed interview was assigned a weight ranging from 0.47 (male users aged 12-17 years) to 2.01 (female users aged 0-1 years). This weighting compensated for the overrepresentation and underrepresentation of specific demographic groups in the CT group relative to the TT group. Weights were calculated as follows: for a given demographic group (eg, male users aged 12-17 years), the proportion of users in the TT group was determined (eg, 1521/100,533, 1.51%). The expected number of users in the CT, assuming the same distribution, was then calculated (eg, 1.51%×16,357=247). The assigned weight for that demographic group was the ratio of the expected number to the observed number of users in the CT group (eg, 247/526=0.47).

#### Statistical Significance

The proportions of users reporting certain medical concepts and self-reported adherence to the recommendation were compared using a chi-square test (with Rao-Scott correction for weighted data), with *P* values adjusted using the Benjamini-Hochberg procedure (false discovery rate [FDR]). Given the large sample size, Cramér *V* effect sizes were calculated alongside *P* values for chi-square comparisons to assess the practical magnitude of observed differences. The number of reported evidence items and interview duration were compared using a survey-weighted Wilcoxon rank-sum test. All tests were interpreted with a significance level of .05. Calculations were performed using Python (version 3.12.13; Python Software Foundation) with the *SciPy* (version 1.16.3) and *statsmodels* (version 0.14.6) packages. Survey-weighted analyses (Rao-Scott corrected chi-square tests and survey-weighted Wilcoxon rank-sum tests) were conducted in R (version 4.6.0; R Foundation for Statistical Computing) using the survey package (version 4.5), interfaced with Python via *rpy2* (version 3.5.17).

### Sentiment Analysis—CT Only

Users’ sentiment was classified using the Gemini 2.5 Flash (Alphabet Inc) LLM by prompting it to describe the user’s sentiment toward the CT tool, rather than general emotions (eg, sadness or frustration with the medical problem the user may have). For each CT encounter, the model was run twice: once on the first 3 user-tool exchanges and subsequently on the entire conversation. Gemini returned a single label—positive, neutral, or negative—together with a rationale for the labeled sentiment. The definitions of the labels were held identical for positive (shows a positive attitude toward the tool by thanking the assistant, asking follow-up questions, or giving direct positive feedback) and negative (shows a negative attitude toward the tool, insults it, or tries to exploit it) sentiment at both time points. The neutral category was defined more narrowly at opening (does not show any attitude) than at closing, where it additionally encompassed plain yes-or-no answers, selection from presented options, and acceptance or rejection of the triage recommendation, reflecting the response formats characteristic of the later dialogue stages. The full prompts are provided in Multimedia Appendix 1.

### Ethical Considerations

This retrospective analysis used anonymized, fully deidentified, preexisting interaction logs from completed triage encounters. No identifiable personal data were included in the analytic dataset, no reidentification was attempted, and no participant contact or intervention occurred. The CT data analyzed in this study were derived from usability-study logs, and the analysis was conducted retrospectively. Given the retrospective design, the use of anonymized or deidentified data, and the absence of participant contact or intervention, formal ethics review was not sought. Under Polish law, formal ethics committee approval is required only for studies meeting the definition of a “medical experiment” under Article 29 of the Act of December 5, 1996, on the Professions of Physician and Dentist. As this study involved no participant contact, no clinical or experimental intervention, and no testing or implementation of new treatments, devices, or technologies on human subjects, it does not meet this definition and was therefore exempt from ethics committee review.

## Results

### Response Volume and User Demographics

A total of 116,890 distinct encounters were completed during the 28-week study period, with 100,533 (86%) encounters recorded in TT and 16,357 (14%) encounters recorded in CT. The 2 groups differed significantly by sex and age (*P*<.001). TT users were predominantly female (71,039/100,533, 71%), whereas CT users had a more balanced sex profile, with 64% (10,402/16,357) female users, suggesting a trend toward a more even sex (male/female) distribution. Although the 18 to 29 years age group was the most common in both modalities, the distribution across age categories diverged markedly. TT was heavily concentrated among younger adults, with more than half of users aged 18 to 29 years, whereas CT showed a broader age spread, with substantial use among adolescents (aged 12-17 years: n=1911, 11.7% vs n=6393, 6.4%), middle-aged adults (aged 30-44 years: n=4167, 25.5% vs n=23,728, 23.6%), and older users (aged ≥45 years: n=3258, 19.9% vs n=16,485, 16.4%), as shown in Table 1. In other words, TT users were concentrated among young adults, whereas CT users were more demographically diverse across both age and sex. All encounters were completed in English, but information about the geographical location of users was not collected.

**Table 1:** Age distribution of users by triage modality (traditional triage vs conversational triage) based on raw, unweighted data.

Age group (years)	Conversational triage tool (n=16,357), n (%)	Traditional triage tool (n=100,533), n (%)	Between-group difference, absolute difference (relative difference)^a^	*P* value^b^	Cramér *V* effect size
0-1	26 (0.2)	271 (0.3)	−0.1 (−41)	.02	0.007
2-3	99 (0.6)	693 (0.7)	−0.1 (−12.2)	.24	0.003
4-11	271 (1.6)	1335 (1.3)	0.3 (24.8)	<.001	0.010
12-17	1911 (11.7)	6393 (6.4)	5.3 (83.7)	<.001	0.072
18-29	6625 (40.5)	51,628 (51.4)	−10.9 (−21.1)	<.001	0.075
30-44	4167 (25.5)	23,728 (23.6)	1.9 (7.9)	<.001	0.015
45-59	2167 (13.2)	11,144 (11.1)	2.2 (19.5)	<.001	0.024
60-74	896 (5.5)	4310 (4.3)	1.2 (27.8)	<.001	0.020
≥75	195 (1.2)	1031 (1)	0.2 (16.2)	.06	0.006

^a^Absolute differences are expressed in percentage points, while relative differences are expressed in percentages.

^b^*P* values were calculated using the chi-square test.

### Interaction Patterns

Although individual user time zones were not recorded, certain use patterns across both tools were observed. A limitation of the current analysis is that time stamps may not accurately reflect real-world user behavior within their local environments. In terms of the day of the week, the highest traffic, defined by the number of sessions completed, occurred on Mondays and Tuesdays. Specifically, the total number of encounters on Mondays was 15,627 (15.5%) for TT and 2555 (15.6%) for CT. The lowest traffic was observed on Saturdays for both tools (13,057/100,533, 13% for TT and 2135/16,357, 13.1% for CT). Regarding times of day, the highest traffic was between 4:00 PM and 10:00 PM UTC and the lowest was between 00:00 and 11:00 AM UTC for both tools. Notably, CT was associated with significantly longer median session durations (8 minutes 21 seconds, IQR 6 minutes 10 seconds-11 minutes 51 seconds) than TT (4 minutes 25 seconds, IQR 3 minutes 14 seconds-6 minutes 22 seconds; *P*<.001), and a lower completion rate—54% (16,357/30,491) for CT and 73% (100,533/137,676) for TT. The lower completion rate in CT suggests that the analyzed CT sample may represent a more engaged subset of the initial user base, which should be considered when interpreting between-group differences. Although users initially reported the same median number of chief complaints in both tools (4), CT interviews collected more medical evidence items overall (symptoms and risk factors; median 36, IQR 31-43, vs 32, IQR 27-38; *P*<.001).

### Clinical Characteristics Reported by the Users

Across both tools, the most frequently declared initial, or chief, complaints were fatigue, abdominal pain, and headache. Relative frequencies, however, were consistently lower in CT than in TT for most of the top chief complaint symptoms: fatigue, 18.1% (2966/16,357) vs 23.5% (23,635/100,533); abdominal pain, 18% (2940/16,357) vs 24% (24,111/100,533); headache, 17% (2785/16,357) vs 23.4% (23,478/100,533; all *P*<.001). This pattern may reflect the broader range of evidence captured in CT, spreading cases across a broader set of symptoms. Mental health chief complaints showed no clear pattern across CT and TT. However, overall mental health–related symptoms were more frequently reported during the interview with CT (rather than as initial or chief complaints, but as evidence reported during the interview), as shown in Table 2: history of a stressful situation, 13.3% (2179/16,357) vs 1% (963/100,533); anxiety states, 15.5% (2533/16,357) vs 13.5% (13,592/100,533); trauma or stressor-related disturbance, 10.4% (1702/16,357) vs 1.7% (1728/100,533); depressive symptoms, 8.5% (1390/16,357) vs 6.2% (6212/100,533); and sleep disturbances, 12.4% (2030/16,357) vs 9.9% (9979/100,533; all *P*<.001). Although these differences reached statistical significance, effect sizes were weak across all comparisons (Cramér *V* range 0.001-0.265), indicating that the practical magnitude of the differences should be interpreted with caution. A notable exception was suicidal thoughts or intent, which was reported comparably frequently in both tools (373/16,357, 2.3% in CT vs 2515/100,533, 2.5% in TT; *P*=.12). Overall, CT encounters included more reported psychosocial context and active mood symptoms. Prior to the main assessment, the CT interface includes an open-ended question inviting users to report any recent lifestyle changes or additional relevant context; in contrast, TT users could only report such information by voluntarily listing it as a chief complaint. This structural difference likely accounts for the higher reporting of psychosocial symptoms in the CT group. Moreover, dermatological changes were more frequently elicited as evidence in CT than in TT (2188/16,357, 13.4% vs 10,661/100,533, 10.6%; a difference of 2.8 percentage points; *P*<.001). This represents a relative increase of more than one-quarter in CT and may reflect differences in how such issues are described in natural language vs selected through predetermined options.

**Table 2:** Subset of reported clinical evidence stratified by modality (chief complaints, symptoms reported during interview, and risk factors)^a^.

Clinical evidence items	Total occurrences^b^ in conversational triage (n=16,357), n (%)	Total occurrences in traditional triage (n=100,533), n (%)	Between-group difference, absolute difference (relative difference)^c^	*P* value^d^	Cramér *V* effect size
Fatigue	7868 (48.1)	48,439 (48.2)	−0.1 (−0.2)	.86	0.001
Nausea	4658 (28.5)	31,329 (31.2)	−2.7 (−8.6)	<.001	0.020
Headache	4465 (27.3)	31,309 (31.1)	−3.8 (−12.3)	<.001	0.029
Abdominal pain and tenderness	4199 (25.7)	30,044 (29.9)	−4.2 (−14.1)	<.001	0.032
Diminished appetite	3302 (20.2)	21,625 (21.5)	−1.3 (−6.2)	<.001	0.011
Anxiety states	2533 (15.5)	13,592 (13.5)	2.0 (14.5)	<.001	0.020
Dizziness	2385 (14.6)	16,186 (16.1)	−1.5 (−9.4)	<.001	0.014
Bloating	2257 (13.8)	14,753 (14.7)	−0.9 (−6)	.007	0.009
Dermatological changes	2188 (13.4)	10,661 (10.6)	2.8 (26.1)	<.001	0.031
History of a stressful situation	2179 (13.3)	963 (1)	12.4 (1290.7)	<.001	0.265
Reduced mobility of body parts	2169 (13.3)	17,658 (17.6)	−4.3 (−24.5)	<.001	0.040
Sleep disturbances	2030 (12.4)	9979 (9.9)	2.5 (25)	<.001	0.028
Joint pain	1992 (12.2)	13,173 (13.1)	−0.9 (−7.1)	.002	0.010
Fever	1932 (11.8)	12,119 (12.1)	−0.2 (−2)	.41	0.003
Overweight and obesity	1879 (11.5)	27,729 (27.6)	−16.1 (−58.4)	<.001	0.128
Trauma or stressor-related disturbance	1702 (10.4)	1728 (1.7)	8.7 (505.2)	<.001	0.178
Depressive symptoms	1390 (8.5)	6212 (6.2)	2.3 (37.5)	<.001	0.033
Suicidal thoughts or intent	373 (2.3)	2515 (2.5)	−0.22 (−8.8)	.12	0.005

^a^Counts are poststratification weighted frequencies rounded to the nearest integer; percentages are weighted proportions of the weighted group totals, which are calibrated to equal the unweighted sample sizes.

^b^Total occurrences refers to the number of encounters in which the item was recorded at any point in the encounter—as a chief complaint, as evidence elicited during the interview, or as a risk factor—divided by the total encounters in that modality. These differ from the chief complaint frequencies reported in the text, which count only items reported as the initial (chief) complaint.

^c^Absolute differences are expressed in percentage points, while relative differences are expressed in percentages.

^d^*P* values were calculated using the Rao-Scott chi-square test and adjusted for multiple comparisons using the Benjamini-Hochberg false discovery rate procedure.

### Posttriage Intent

A higher proportion of CT users reported their intended actions after triage (5104/16,357, 31.2% completed the intent survey vs 5968/100,533, 5.9% in TT; *P*<.001). Among respondents, overall self-reported adherence to triage recommendations was higher in CT than in TT (1749/5104, 34.3% vs 1740/5968, 29.2%; *P*<.001). Importantly, the greatest differences emerged at the extremes of acuity, as shown in Table 3: self-reported intended adherence to self-care advice was substantially higher among CT respondents (534/625, 85.4% vs 325/525, 61.9%), while adherence to urgent recommendations also increased, more than doubling for ER guidance (171/723, 23.7% vs 100/949, 10.5%) and for ambulance calls (39/339, 11.5% vs 27/554, 4.9%; all *P*<.001). By contrast, TT respondents were slightly more likely to follow routine consultation advice (705/2181, 32.3% for TT vs 553/1941, 28.5%, for CT; *P*=.01), and adherence to 24-hour consultation advice did not differ significantly (583/1759, 33.1% for TT vs 452/1476, 30.6%, for CT; *P*=.13). Overall, CT was associated with substantially more postvisit feedback completion, and among respondents, self-reported intended adherence was higher at both the lowest and highest acuity levels. Because survey completion differed substantially between modalities, these adherence comparisons should be interpreted as respondent-subset findings and may be affected by nonresponse bias.

**Table 3:** Self-reported intended adherence to triage recommendations by acuity level and triage modality^a^.

Triage levels	Intended adherence to conversational triage, n (%)	Intended adherence to traditional triage, n (%)	Between-group difference, absolute difference (relative difference)^b^	*P* value^c^	Cramér *V* effect size
1. Self-care	534/625 (85.4)	325/525 (61.9)	23.5 (38)	<.001	0.267
2. Consultation	553/1941 (28.5)	705/2181 (32.3)	−3.8 (−11.9)	.01	0.041
3. 24-hour consultation	452/1476 (30.6)	583/1759 (33.1)	−2.5 (−7.6)	.13	0.026
4. Emergency room	171/723 (23.7)	100/949 (10.5)	13.1 (124.5)	<.001	0.174
5. Emergency ambulance	39/339 (11.5)	27/554 (4.9)	6.6 (136.1)	<.001	0.117
Total^d^	1749/5104 (34.3)	1740/5968 (29.2)	5.1 (17.5)	<.001	0.055

^a^Counts are poststratification weighted frequencies rounded to the nearest integer; percentages are weighted proportions of the weighted group totals, which are calibrated to equal the unweighted sample sizes.

^b^Absolute differences are expressed in percentage points, while relative differences are expressed in percentages.

^c^*P* values were calculated using the Rao-Scott chi-square test and adjusted for multiple comparisons using the Benjamini-Hochberg false discovery rate procedure.

^d^Total number of respondents differs because these analyses include only users who completed the voluntary posttriage intent survey. Each respondent is counted only at the acuity level they were assigned, which was unevenly distributed across the 5 levels.

### User Sentiment (CT Users Only)

Sentiment analysis was restricted to the CT group, as the TT interface did not support free-text expression; the absence of a comparable TT baseline precludes attribution of observed sentiment patterns solely to the conversational modality. Sentiment labels were generated by a single AI model using an iteratively refined prompt intentionally calibrated to treat postassessment follow-up questions as indicative of positive sentiment in the absence of explicit user frustration, which may result in modest overestimation of positive sentiment; overall label accuracy appeared satisfactory after a manual review of representative examples. However, formal human validation was not performed, which represents a methodological limitation to be addressed in future work. Sentiment analysis of CT dialogues showed predominantly neutral openings (96.9%). By conversation end, positivity increased more than 14-fold (218/16,316, 1.3% to 3053/16,316, 18.7%) and negativity more than 4-fold (283/16,316, 1.7% to 1252/16,316, 7.7%), with absolute gains of 17.4 percentage points and 6.0 percentage points, respectively. Thus, approximately three-quarters of users who left neutrality did so toward a positive sentiment. The ratio of positive to negative sentiment reversed and widened from 0.76 at opening to 2.43 by close, indicating that among users who formed a view by the end of the dialogue, positive sentiment predominated while most users remained neutral, as shown in Figure 4.

**Figure 4:**
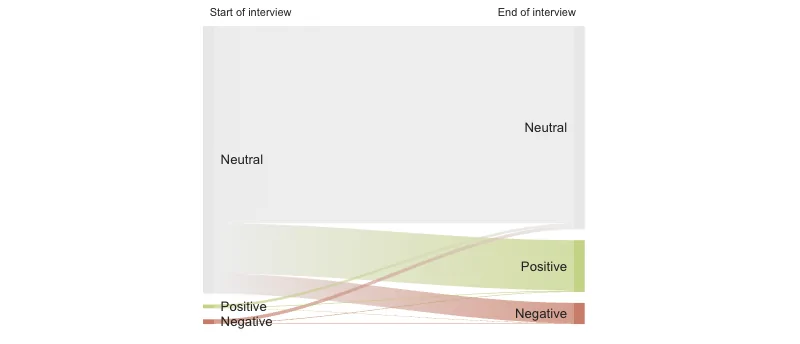
Change in user sentiment from the start to the end of conversational triage sessions, as labeled by a large language model (Gemini 2.5 Flash).

## Discussion

### Use Patterns and Demographics

TT, as the longer-standing legacy tool, recorded more interviews during the studied period, with 100,533 (86%) completed encounters compared with 16,357 (14%) for CT, with CT users having more evenly distributed demographics (age and sex). TT users were more often female (n=71,039, 71% vs n=10,402, 64% in CT) and had a more clustered age distribution, whereas CT showed a broader spread across age groups, with users aged >30 years being more common in CT. Therefore, CT was associated with a broader user profile, including relatively higher proportions of male and older users, suggesting potentially greater accessibility across demographic groups. This observation is consistent with recent survey evidence showing that uptake, perceptions, and experiences of LLMs in health care may vary across user groups, underscoring the importance of evaluating demographic patterns, engagement, and usability in real-world deployments [18,19]. Interaction patterns were broadly similar by time of day and day of the week, yet CT sessions were substantially longer at 8 minutes 21 seconds (IQR 6 minutes 10 seconds to 11 minutes 51 seconds) vs 4 minutes 25 seconds median (IQR 3 minutes 14 seconds to 6 minutes 22 seconds) for TT. Although part of the reason for the difference in durations might be technical (eg, CT latency to generate subsequent messages was higher—by 88 seconds across the entire interview duration), the longer session may reflect both technical factors and a more detailed conversational assessment, as CT encounters included more total evidence items. Although longer consultations correlate with satisfaction in traditional care settings [20], this relationship cannot be assumed in digital triage. In this study, CT was associated with greater survey completion, higher self-reported intended adherence among respondents, and a shift in sentiment from neutral toward positive. However, CT also had a substantially lower interview completion rate than TT (16,357/30,491, 54% vs 100,533/137,676, 73%), meaning that a considerable proportion of CT users did not reach the final recommendation; this pattern of higher postcompletion engagement alongside a greater dropout rate suggests an element of survivorship bias in the CT sample. Because response rates differed substantially between CT and TT, adherence findings should be interpreted as respondent-subset results and may reflect differences in who completed the survey. Taken together, these findings suggest that CT was associated with deeper engagement among the subset of users who completed the interview, while causal conclusions about satisfaction, trust, or adherence remain limited.

### Clinical Presentations

When it comes to the clinical characteristics reported by users, the observed differences may be related to tool-specific patterns of interface-mediated disclosure and engagement, rather than differences in medical logic, because both modalities share the same probabilistic engine and medical knowledge base. First, CT encounters included more frequent reporting of psychosocial context: users volunteered more mental health evidence during the interview (eg, anxiety states: n=2533, 15.5% vs n=13,592, 13.5%; depressive symptoms: n=1390, 8.5% vs n=6212, 6.2%; all *P*<.001). Importantly, CT did not start with higher mental health chief complaint reporting, suggesting that the observed differences do not appear to reflect only reclassification of visit motives, but also greater reporting of additional context during the dialogue. This pattern is consistent with the possibility that free-text dialogue may lower barriers to sensitive or contextual information sharing. Second, the higher number of evidence items reported alongside longer sessions indicates that the conversational modality was associated with iterative clarification and expansion of history elements beyond the initial complaint set. These findings are consistent with evidence that conversational LLM-based tools can convey empathy and encourage sharing of sensitive information [11,21].

### Clinical and Operational Implications of Posttriage Intent

A notable finding was the substantially higher completion of the voluntary posttriage intent survey among CT users. There was more than a 5-fold increase in posttriage intent reporting with CT compared with TT (5104/16,357, 31.2% vs 5968/100,533, 5.9%; *P*<.001). Among survey respondents, CT also showed higher overall self-reported intended alignment with recommendations. However, because survey completion differed substantially between modalities, these adherence comparisons are vulnerable to nonresponse bias and should be interpreted as applying only to respondents. The most significant differences were at the two ends of the acuity spectrum: self-care adherence (534/625, 85.4% vs 325/525, 61.9% for CT and TT, respectively) and emergency and ambulance adherence (171/723, 23.7% vs 100/949, 10.5%; 39/339, 11.5% vs 27/554, 4.9%; all *P*<.001). Although TT users more often followed routine consultation advice, the magnitude of the difference was less pronounced (553/1941, 28.5% vs 705/2181, 32.3% for CT and TT, respectively; *P*=.01). This is particularly notable given prior research showing that patients tend to place relatively low trust in LLMs compared with traditional search engines and website-based health information [22]. One possible explanation for the higher self-reported intended adherence observed in CT may be that the LLM component was combined with more explainable and validated technology, potentially mitigating skepticism. However, further study is needed to confirm this mechanism.

These findings suggest that, among survey respondents, CT may support acceptance of recommendations at both ends of the acuity spectrum, including self-care advice and emergency care guidance. Increased intended adherence at the lowest and highest acuity triage recommendations has potential implications for safety and system efficiency. If confirmed prospectively, greater adherence to self-care advice could reduce unnecessary service use, while greater adherence to emergency and ambulance guidance could shorten delays for high-risk cases.

From an implementation perspective, these findings suggest that CT may be most useful when health systems aim to support users who benefit from free-text symptom description, clarification, and more detailed contextual information gathering. Structured questionnaire-based triage may remain preferable when speed, standardization, and lower interaction burden are priorities; in many digital front door settings, both approaches could be offered in parallel to accommodate different user preferences, acuity contexts, and operational workflows.

Finally, sentiment analysis showed predominantly neutral openings (96.9%); by conversation end, positivity rose to 18.7% and negativity to 7.7%, with the positive-to-negative ratio reversing from 0.76 at opening to 2.43 at close. Although not a direct measure of satisfaction or trust, this widening distribution—skewed toward positivity—suggests a favorable user outlook. This pattern aligns with the higher posttriage intent survey completion rate and, among survey respondents, higher self-reported intended adherence to posttriage recommendations.

### Study Limitations

This study has several important limitations. First, it is a retrospective observational analysis of self-selected users on a publicly available website, introducing selection bias. Users who chose a conversational interface may differ systematically from those who chose the TT in ways that could influence both engagement and self-reported intended adherence (eg, comfort with free text, digital and health literacy, symptom severity). Second, the groups were markedly imbalanced in size (100,533/116,890, 86% TT encounters vs 16,357/116,890, 14% CT encounters), which may have introduced unequal precision of estimates across modalities. Similarly, the proportion of users completing the assessment was lower in CT, introducing potential survivorship bias. In addition, although demographic weighting was applied to mitigate composition differences between CT and TT, residual confounding by unmeasured factors (eg, comorbidity, prior triage experience, cultural or geographical background) could not be adjusted. Furthermore, the accuracy of LLM-driven concept extraction and its downstream effect on triage outputs represent an important area for future investigation that falls outside the scope of the present study. Third, adherence was measured via a voluntary posttriage intent survey. Response rates differed significantly between the tools (5104/16,357, 31.2% CT vs 5968/100,533, 5.9% TT), so adherence estimates reflect the subset who responded and may be subject to nonresponse bias. Moreover, posttriage intent is not equivalent to observed behavior or clinical outcomes. Because geographic location was not collected, we could not assess whether regional differences in access or costs influenced intended adherence to high-acuity recommendations. Additionally, the platform did not technically restrict users from submitting multiple responses; however, given the unintended nature of repeated use, the proportion of duplicate submissions is expected to be negligible. Fourth, the sentiment analysis—limited to positive, neutral, or negative labels—used a single LLM (Gemini 2.5 Flash), was limited to CT assessment, and provided a coarse proxy for affect that might be open to misinterpretation or bias. In particular, the sentiment definitions provided to the labeling model classified follow-up questions as indicative of positive sentiment; however, because the conversational interface is inherently designed to elicit dialogue, follow-up questions may equally reflect a practical need for clarification rather than a positive attitude; this definitional choice may have inflated the observed positive sentiment estimates, and results should be interpreted with this potential bias in mind. However, although this operational definition likely inflates absolute positivity scores, we argue that it still serves as a meaningful proxy for active user engagement and conversational persistence, contrasting sharply with immediate user friction or platform abandonment. Nonetheless, future research should use more nuanced classification rubrics that cleanly separate transactional or clarifying inquiries from explicit affective satisfaction. In addition, the neutral category was defined more broadly at conversation close than at opening (Multimedia Appendix 1), so the measurement rubric was not fully constant across time points. However, because the broader closing definition classifies more responses as neutral, this discrepancy is conservative with respect to the principal finding of movement away from neutrality: the reported increases in positive and negative sentiment are, if anything, underestimated. Note that this conservatism applies to the magnitude of the shift; its effect, if any, on the balance between positive and negative labels cannot be determined from the rubric difference alone. Furthermore, because the TT interface did not support free-text input, the lack of a baseline comparison means that these sentiment trends should not be linked solely to the conversational interface. Fifth, the different modalities had different user interfaces and therefore some of the observed effects may reflect user interface and user experience differences, which this study cannot disentangle. Finally, although both modalities are based on certified medical device technology, the individual triage interviews were not retrospectively reviewed by clinicians. Consequently, we could not assess case-level recommendation accuracy or clinical outcomes, and the adherence findings should be interpreted as self-reported intended alignment with recommendations rather than observed care-seeking behavior.

### Conclusions

This real-world comparative study found that an LLM-enabled CT interface layered on a validated probabilistic engine was associated with richer clinical context than a traditional structured AI questionnaire, particularly for mental health and dermatological symptoms. The conversational interface was also used by a more demographically diverse population and, among voluntary posttriage survey respondents, was associated with higher self-reported intended adherence to recommended care, especially for self-care and high-acuity recommendations. These findings suggest that an LLM-enabled CT interface may support greater user engagement and more comprehensive information gathering in digital front door settings, with potential implications for care navigation. This highlights the potential role of LLM-enabled, clinically validated CT in digital front door strategies and care navigation. Given the observational design and other study limitations, further prospective research is needed to evaluate causal effects, assess the impact on real-world care-seeking behavior, and optimize CT for safety, completeness, equity, and user experience.

## Appendix

Multimedia Appendix 1 Verbatim prompt used for sentiment analysis with Gemini 2.5 Flash.

## Abbreviations

CT: conversational triage
ER: emergency room
FDA: Food and Drug Administration
FDR: false discovery rate
LLM: large language model
TT: traditional triage
UTC: Coordinated Universal Time

## Data Availability

The data analyzed in this study are not publicly available because they consist of proprietary, deidentified interaction logs from the Symptomate platform, which is owned by Infermedica. The aggregated data supporting the findings of this study are presented within the manuscript. Additional data may be made available from the corresponding author upon reasonable request and subject to approval by Infermedica and applicable privacy, legal, and commercial restrictions.
