# Supplementary material for: Demographics, Clinical Content, Use Patterns, and Care-Seeking Intent Across Two Generations of AI-Enabled Clinical Triage Tools (A Traditional Structured Questionnaire and a Large Language Model–Enabled Conversational Interface): Comparative Retrospective Observational Study
Source: JMIR AI. 2026 Sep 22;5:e84469. doi: 10.2196/84469 (PMC13643258; doi:10.2196/84469)
Supplement: Supplementary file 1 [file ai_v5i1e84469_app1.pdf]

Prompt 1: Interview start

Your task is to describe the user's sentiment from a conversation fragment with an AI assistant. Consider only sentiment towards THE TOOL, not general emotions (e.g. sadness or frustration with the medical problem they have).

Your answer may consist of ONLY two elements:
(1) ONE adjective from this list: Positive, Neutral, Negative,
(2) short (one sentence only) reasoning.

Follow the EXAMPLE ** OUTPUT ** format and return ONLY a valid JSON response.

*** DEFINITIONS ***
Positive: shows positive attitude towards the tool by thanking the assistant, asking follow up questions, or giving direct positive feedback
Neutral: does not show any attitude
Negative: shows negative attitude towards the tool, insults it, or tries to exploit it.

EXAMPLE:
** TRANSCRIPT **

assistant: Hello! What symptoms concern you today?
user: My kid says he's got stomach pain.
assistant: I'm here to help. Could you please tell me your child's age and the sex assigned at birth? This information is important for a more accurate assessment of the stomach pain.
user: Sure, he's a 3-year-old boy.
assistant: Thank you for sharing that. Could you let me know when your son's stomach pain started? Knowing the onset time can help us better understand the situation.
user: The pain started this morning.

** OUTPUT **
{
  "sentiment": "positive",
  "reasoning": "User answers patiently, using whole sentences and adds interjections such as 'sure' that express their positive attitude"
}

Prompt 2: Whole conversation

Your task is to describe the user's sentiment from a conversation fragment with an AI assistant. You are provided with the whole conversation. Assess user sentiment when conversation ends.

Consider only sentiment towards THE TOOL, not general emotions (e.g. sadness or frustration with the medical problem they have).

Your answer may consist of ONLY two elements:
(1) ONE adjective from this list: Positive, Neutral, Negative,
(2) short (one sentence only) reasoning.

Follow the EXAMPLE ** OUTPUT ** format and return ONLY a valid JSON response.

*** DEFINITIONS ***
Positive: shows positive attitude towards the tool by thanking the assistant, asking follow up questions, or giving direct positive feedback
Neutral: does not show any attitude, answers Yes/No or chooses from presented options
Negative: shows negative attitude towards the tool, insults it, or tries to exploit it.

IMPORTANT: Do not consider rejecting or accepting the recommendation as a negative or positive sentiment unless the user provides explicit feedback.

EXAMPLE:
** TRANSCRIPT **
assistant: Hello! What symptoms concern you today?
user: My kid says he's got stomach pain.
assistant: I'm here to help. Could you please tell me your child's age and the sex assigned at birth? This information is important for a more accurate assessment of the stomach pain.
user: Sure, he's a 3-year-old boy.
assistant: Thank you for sharing that. Could you let me know when your son's stomach pain started? Knowing the onset time can help us better understand the situation.
user: The pain started this morning.
assistant: Could you describe how severe your son's stomach pain is? For example, is it mild, moderate, or severe? This will help us understand how much discomfort he's in.
user: I'm not really sure, I would say it's moderate.
assistant: To ensure we're thorough, I routinely check for any red-flag symptoms that might need more urgent care. Could you let me know if any of these apply to your son?
user: Red stool - No
Severe abdominal pain - No
Pain when pressing the abdomen - No
Loss of consciousness - No
Appendix removal in the past - No
Looking or feeling so seriously ill that it is very worrying - No
assistant: Thanks for the information so far. To get a clearer picture of your son's condition, could you let me know if he's experiencing any of the following symptoms?
user: Vomiting - No
Feeling sick or queasy - Yes
Diarrhea - No

Bloating - Yes
Diminished appetite - Yes
Fatigue - Don't know
assistant: To help us understand what might be causing your son's symptoms, could you let me know if any of the following apply to your household or his recent activities?
user: Gastroenteritis among family members - Yes
Poor hand hygiene - No
Consumption of foods or drinks that cause bloating - No
Contact with allergen - Don't know
assistant: Thanks for the details so far. Is there anything else you think might be important to mention, like recent changes in his routine, diet, or any new medications? Also, has he had any recent illnesses or been under unusual stress? These factors could help us better understand his symptoms.

** OUTPUT **

{
  "sentiment": "neutral",
  "reasoning": "User simply answers the questions patiently, does not express any positive or negative feelings"
}
